# Biochemical Background in Mitochondria Affects 2HG Production by IDH2 and ADHFE1 in Breast Carcinoma

**DOI:** 10.3390/cancers13071709

**Published:** 2021-04-04

**Authors:** Jitka Špačková, Klára Gotvaldová, Aleš Dvořák, Alexandra Urbančoková, Kateřina Pospíšilová, David Větvička, Alberto Leguina-Ruzzi, Petra Tesařová, Libor Vítek, Petr Ježek, Katarína Smolková

**Affiliations:** 1 Institute of Physiology of the Czech Academy of Sciences, Laboratory of Mitochondrial Physiology, 142 20 Prague, Czech Republic; jitka.spackova@fgu.cas.cz (J.Š.); klara.gotvaldova@fgu.cas.cz (K.G.); alesh.dvorak@gmail.com (A.D.); alexandra.urbancokova@fgu.cas.cz (A.U.); AlbertoAndres.LeguinaRuzzi@fgu.cas.cz (A.L.-R.); petr.jezek@fgu.cas.cz (P.J.); 2 Institute of Medical Biochemistry and Laboratory Diagnostics of the General University Hospital and the First Faculty of Medicine, Charles University, 121 08 Prague, Czech Republic; pospisilova.kp@gmail.com (K.P.); vitek@cesnet.cz (L.V.); 3 Institute of Biophysics and Informatics, the First Faculty of Medicine, Charles University, 120 00 Prague, Czech Republic; david.vetvicka@gmail.com; 4 Department of Oncology, the General University Hospital and The First Faculty of Medicine, 128 08 Prague, Czech Republic; tesarova.petra@seznam.cz; 5 4 th Department of Internal Medicine, the General University Hospital and the First Faculty of Medicine, Charles University, 128 08 Prague , Czech Republic

**Keywords:** IDH2, 2HG, breast carcinoma

## Abstract

**Simple Summary:**

2-hydroxyglutarate (2HG) is a metabolite resembling normal cell metabolite 2-oxoglutarate (2OG), however, its accumulation in cells might lead to amplification of processes in cancer development. R-2HG is a product or bi-product of several metabolic enzymes, including mitochondrial ones. We investigated whether production of mitochondrial 2HG is elevated in breast cancer cell lines and identified active competition for initial substrate, 2OG, between enzymes isocitrate dehydrogenase IDH2 and alcohol dehydrogenase ADHFE1. We have also investigated possible substrate and cofactor NADPH channeling between the two IDH2 molecules within mitochondria. We characterized several situations when either IDH2 and ADHFE1 produce a non-negligible amount of 2HG, which is then actively exported from cells. This can serve as a clinical application of our findings. We have therefore quantified 2HG levels in the urine of breast carcinoma patients after resection of their tumors and showed a positive correlations between cancer stages and 2HG levels. Note that cancer stages I to IV differ by the existence and severity of metastases. Extension of these findings might help to improve diagnostic approaches of breast carcinoma.

**Abstract:**

Mitochondrial production of 2-hydroxyglutarate (2HG) can be catalyzed by wild-type isocitrate dehydrogenase 2 (IDH2) and alcohol dehydrogenase, iron-containing 1 (ADHFE1). We investigated whether biochemical background and substrate concentration in breast cancer cells promote 2HG production. To estimate its role in 2HG production, we quantified 2HG levels and its enantiomers in breast cancer cells using analytical approaches for metabolomics. By manipulation of mitochondrial substrate fluxes using genetic and pharmacological approaches, we demonstrated the existence of active competition between 2HG producing enzymes, i.e., IDH2 and ADHFE1. Moreover, we showed that distinct fractions of IDH2 enzyme molecules operate in distinct oxido-reductive modes, providing NADPH and producing 2HG simultaneously. We have also detected 2HG release in the urine of breast cancer patients undergoing adjuvant therapy and detected a correlation with stages of breast carcinoma development. In summary, we provide a background for vital mitochondrial production of 2HG in breast cancer cells with outcomes towards cancer biology and possible future diagnosis of breast carcinoma.

## 1. Introduction

Metabolic reprogramming is a general feature of cancer phenotype. There is an accepted consensus that the reason for cancer metabolic changes is to provide biosynthetic precursors during rapid cancer growth, including building blocks, energy intermediates, and reducing equivalents. Therefore, accumulating knowledge on cancer metabolism brings the opportunity not only to better understand cancer biology but also to reveal potential markers for diagnosis and critical targets of cancer therapy [1]. 

Substantial attention concerning research on mitochondrial isocitrate dehydrogenase 2 (IDH2) and cytosolic IDH1 in cancer cells has been devoted to mutant forms of IDHs, representing oncogenes, producing 2-hydroxyglutarate (2HG) [2]. Enantiomers of 2HG (R- and S-) are epigenetically active oncometabolites, which inhibit 2-oxoglutarate (2OG)-dependent dioxygenases (e.g., JMJC, TET2, EGLN2), and, consequently, support cell transformation by restraining differentiation [2,3,4,5]. Elevated concentrations of 2HG can be readily detected in the circulation of oncogenic patients [6,7]. Previously, we have demonstrated that 2HG can be formed by mitochondrial wild-type IDH2 despite the absence of any IDH2/1 mutations in breast cancer cells [8,9,10]. 2HG formation by wild-type IDH2 in breast cancer was also reported by Terunuma [11] and associated with the activity of the Myc oncogene, which augments glutaminolysis, and thus supports also reductive reactions catalyzed by IDH2, i.e., the reductive carboxylation (RC) into isocitrate (IC) and reduction of 2OG into 2HG. Besides, hydroxyacid-oxoacid transhydrogenase (alcohol dehydrogenase, iron-containing 1, ADHFE1) is another potential mitochondrial producer of glutamine-derived R-2HG [12,13]. S-2HG enantiomer is formed as a non-canonical product of malate dehydrogenase (MDH) and lactate dehydrogenase [14,15]. Both enantiomers of 2HG are degraded by two specific mitochondrial dehydrogenases, namely L2HGDH and D2HGDH [16].

We assume that the reduction of 2OG into R-2HG by wild-type IDH2 proceeds along with isocitrate by RC. Conditions required for RC and 2HG formation by wild-type IDH2 involve a so-called truncated Krebs cycle, sufficient substrate levels (2OG, formed in glutaminolysis), and co-factor (NADPH) availability. Several studies indicated that a low citrate/2OG ratio is an ultimate parameter regulating the RC in mitochondria [17,18]. 2OG in mitochondria is to a large extent generated by glutaminolysis, the flux of which is regulated by the expression and activity of glutaminolysis enzymes (glutaminase, glutamate dehydrogenase, and mitochondrial transaminases). Moreover, NADPH is also crucial in regulating IDH2 reductive functions. Interestingly, the affinity of IDH2 to NADPH might be higher than to NADP^+^ [19] and therefore reductive function is preferred in the redox settings of the mitochondrial matrix. It has been demonstrated that knockdown of 2OG-dehydrogenase (2OGDH) negatively regulates reductive glutamine utilization [20] because it also suppresses the formation of NADH, which is a substrate for mitochondrial nicotinamide nucleotide transhydrogenase (NNT) in the reaction producing NADPH. It has been also demonstrated that NNT supports reductive glutamine utilization [21]. Therefore, the availability of mitochondrial NADPH must be considered as a factor controlling the IDH2 reductive activity in addition to the 2OG/citrate ratio. To understand mitochondrial 2HG production and reductive metabolism associated with IDH2, it is important to determine the major source of mitochondrial NADPH. 

In this study, we aimed to analyze the conditions that lead to maximum 2HG production by wild-type IDH2 in breast cancer cells and to gather background information regarding cellular 2HG turnover. We hypothesized that the formation of mitochondrial 2HG depends on the biochemical and genetic background, including NADPH and 2OG production, and expression levels of related competing enzymes, such as ADHFE1. Hence, we evaluated 2HG levels in several breast carcinoma cultured cell lines, derived from primary and secondary tumors and we demonstrated that 2HG is actively formed by IDH2 and ADHFE1 and that there is a competition between these two enzymes, which regulate substrate flux in mitochondria. Moreover, 2HG production in vitro was also confirmed in a small clinical study of breast carcinoma patients after tumor resection demonstrating that 2HG levels in breast carcinoma patients are sufficiently high for to be secreted to the extracellular interstitial, to lymph and blood to be finally reflected by e.g., urine levels. 

## 2. Results

### 2.1. HG Production in Breast Cancer Cells

**Figure 1:**
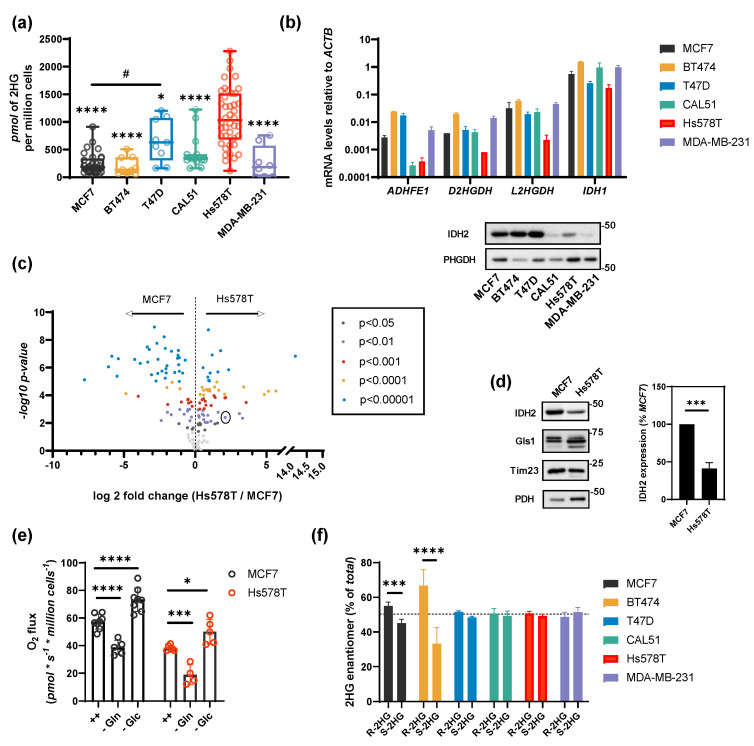
2HG production in breast cancer cells. (**a**) Box plots of 2HG production in breast cancer cell lines. *n* > 8. # *p* < 0.05 MCF7 vs. T47D. One-way ANOVA. (**b**) Quantification of the expression level of genes/proteins related to 2HG production measured by qPCR or western blot. (**c**) Volcano plot of polar metabolites in MCF7 and Hs578T cells. 2HG is indicated by the circle. (**d**) Comparison of IDH2 levels in MCF7 and Hs578T cells. *n* > 3. *t*-test. (**e**) Respiration of MCF7 and Hs578T cells in glucose and glutamine-free media, respectively. *n* > 5. Two-way ANOVA. (**f**) Percent of R- and S-enantiomers of 2HG in breast cancer cell lines. *n* > 3. Two-way ANOVA. * *p* < 0.05, *** *p* < 0.001, **** *p* < 0.0001.

2HG production in mitochondria depends on few enzymes; IDH2, ADHFE1, and MDH. We tested six breast cancer cell lines using gas chromatography coupled with mass spectrometry (GC-MS) and revealed distinct levels of 2HG with maximal production in Hs578T cell line, which paradoxically expresses low levels of IDH2 (Figure 1a,b,d). According to our previous work [8], ^13^C-labeling of 2HG has been observed when we used 1-^13^C-glutamine (IDH2-related flux) and we have demonstrated that 2HG production in Hs578T could be diminished by IDH2 knockdown. In order to understand the relationship between the production of 2HG by IDH2 and substrate flux in mitochondria, we compared cell lines with high vs. low levels of 2HG and the pattern of IDH2 expression, respectively (Figure 1a,b,d), i.e., MCF7 and Hs578T. Different metabolic settings between the respective cell lines were observed also by metabolomics as demonstrated by volcano plot (Figure 1c and Figure S1b), where 2HG was plotted with 0.01 significance and more than four-fold higher 2HG level in Hs578T compared to MCF7 (log2 fold-change of 2.11). Among the six tested breast cancer cell lines, MCF7 and Hs578T cells showed similar survival in glutamine and glucose-free conditions (Figure S1c), and similar respiratory rates (Figure 1e). We subsequently used MCF7 and Hs578T for further experiments. Chiral separation of 2HG enantiomers showed that about 50% of 2HG in Hs578T and MCF7 cells is in R-configuration (Figure 1f), indicating a possible involvement of IDH1, IDH2, PHGDH, or ADHFE1.

### 2.2. Substrate Flux Determines 2HG Production in Breast Cancer Cells

**Figure 2:**
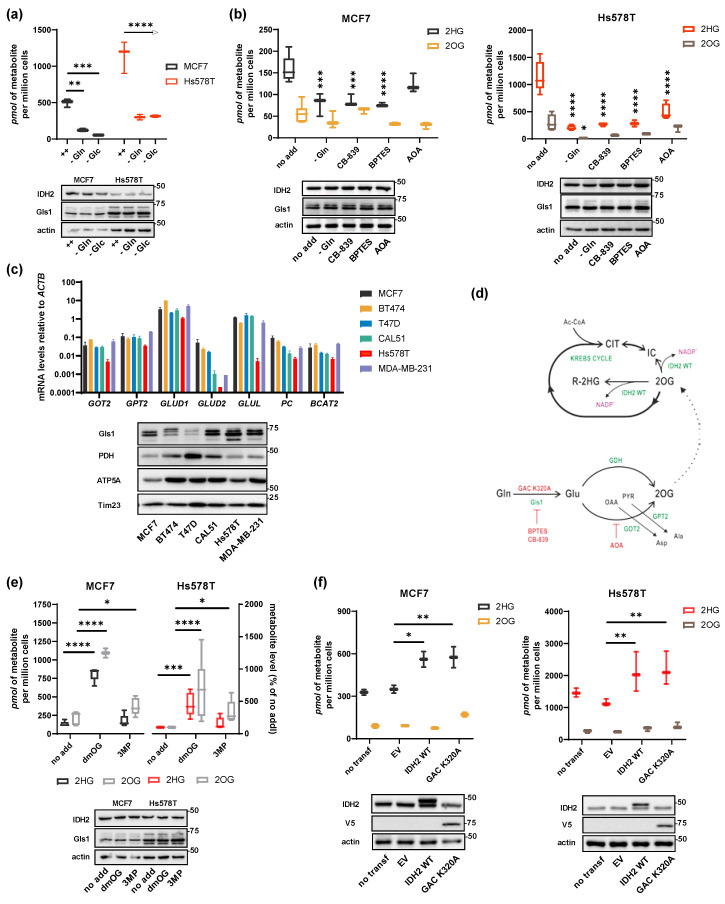
Substrate flux regulates 2HG mitochondrial production. (**a**) 2HG decline in MCF7 and Hs578T cells in glucose and glutamine-free media, respectively. *n* = 3. Two-way ANOVA. (**b**) 2HG and 2OG production after treatment *o/n* with inhibitors of glutaminolysis, i.e., CB-839 (5 µM), BPTES (10 µM), (Aminooxy)acetic acid hemihydrochloride (AOA, 500 µM). *n* > 3. Two-way ANOVA. Western blots depict the expression of IDH2 and Gls1 in parallel samples. (**c**) Quantification of the expression level of genes related to 2HG production measured by qPCR. Gls1 expression assessed by western blot. Expression of mitochondrial markers pyruvate dehydrogenase (PDH), ATP synthase F1 subunit alpha (ATP5A), and mitochondrial import inner membrane translocase subunit Tim23. (**d**) Scheme depicting the glutaminolysis pathway and manipulations used in the experiments. (**e**) 2HG production after addition of dmOG (2 mM, 6 h) and 3-methylpyruvate (2 mM, 6 h) in MCF7 and Hs578T. *n* = 10. Two-way ANOVA. Western blots depict the expression of IDH2 and Gls1 in parallel samples. (**f**) 2HG and 2OG production in cells transfected with IDH2 WT and GAC K320A. *n* = 3. Two-way ANOVA. Western blots depict the expression of IDH2 and GAC (bearing V5-epitope) in parallel samples.* *p* < 0.05, ** *p* < 0.01, *** *p* < 0.001, **** *p* < 0.0001.

Next, we aimed to quantify to what extent glucose and glutamine fluxes contribute to mitochondrial 2HG production in cells expressing low and high levels of IDH2, respectively. First, glutamine or glucose removal from the growing medium effectively decreased 2HG levels in both cell lines (Figure 2a), decreasing also other TCA intermediates, though (Figure S2a). Treatment with inhibitors of glutaminolysis (CB-839, BPTES, and AOA) has consistently lowered the levels of 2HG in all tested cell lines regardless of IDH2 expression level (Figure 2b). These results suggest that glutaminolysis flux creates metabolic precursors for downstream 2HG production. The addition of 2 mM dimethyl-oxoglutarate (dmOG) [22], induced 2HG to a similar extent in both MCF7 and Hs578T cells (over five-fold for dmOG), respectively (Figure 2e). A permeable pyruvate analog 3-methyl-pyruvate (3MP) also induced 2HG production, to the lower extent, though (Figure 2e). To validate the observation that glutaminolysis might ensure the 2OG supply for 2HG synthesis (i.e., IDH2 reaction), we overexpressed the glutaminase C (GAC) superactive mutant K320A (Figure 2d) [23] and analyzed 2HG production in the cell lines with distinct levels of IDH2 (lower in Hs578T than MCF7 cells). Overexpression of GAC K320A resulted in the elevated 2HG production (Figure 2f), indicating that glutaminolysis-produced 2OG is channeled into 2HG. Different ratios of 2HG/2OG in Hs578T and MCF7 might reflect the effectivity of 2OG channeling into 2HG synthesis. As 2OG levels in Hs578T were not elevated after transfection with GAC K320A (Figure 2f, right), we presume that 2OG is effectively channeled into 2HG synthesis, while the level of 2OG is saturated for relatively low 2HG production capacity in MCF7 cells, so 2HG producing enzymes are limiting factor in MCF7. We conclude that glutaminolysis is the dominant carbon contributor to 2HG synthesis in mitochondria and that the efficiency of 2HG production depends on the effectiveness of the substrate channeling into 2HG-producing reactions.

### 2.3. Shared 2HG Production by ADHFE1 and IDH2 in Breast Cancer Cells

**Figure 3:**
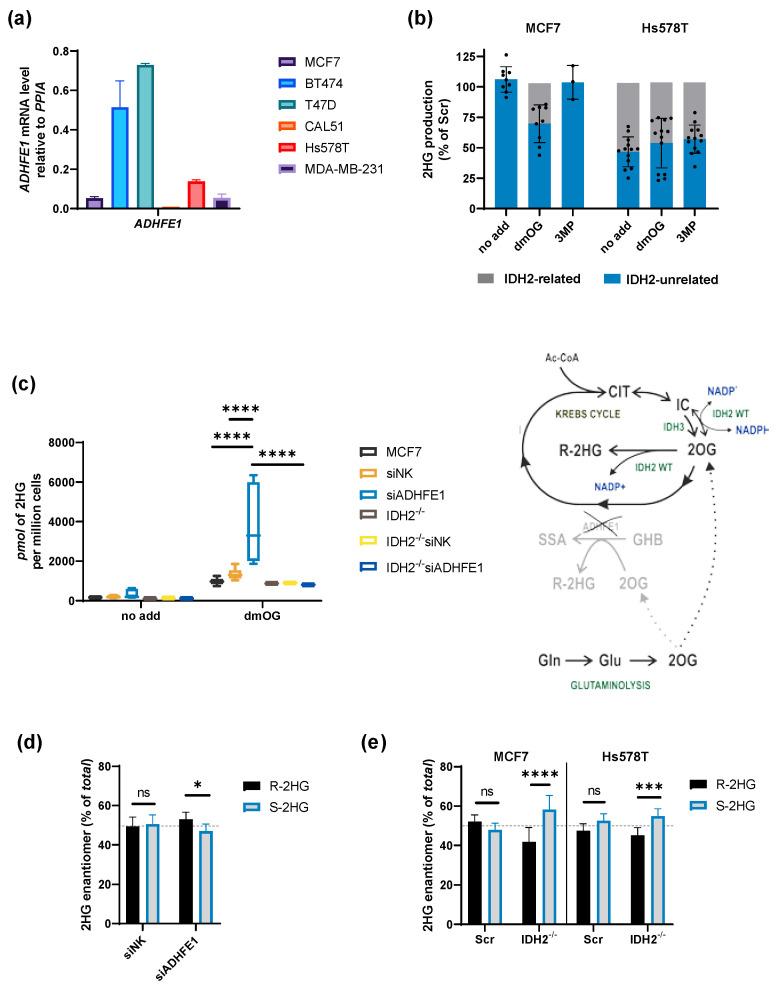
2HG production by IDH2 and ADHFE1. (**a**) Quantification of the expression level of ADHFE1 measured by qPCR. (**b**) IDH2-related and IDH2-unrelated portions of 2HG were produced in MCF7 and Hs578T cells, respectively. IDH2-related portions were calculated indirectly comparing IDH2^+/+^ (scrambled, Scr) and IDH2^−/−^ cell lines, respectively. See also Figure S3a,b for actual experiments. (**c**) 2HG levels in MCF7 cells, transfected with non-targeting siRNA (siNK) and siRNA targeting ADHFE1 (siADHFE1), non-treated and treated with dmOG (2 mM, 6 h). *n* = 9. Two-way ANOVA. Right: scheme depicting the experimental design, i.e., substrate flow after knockdown of ADHFE1 using siRNA. GHB stands for *gamma*-Hydroxybutyric acid and SSA for succinyl-semialdehyde. (**d**) Per-cent of R-HG and S-2HG in MCF7 cells, transfected with non-targeting siRNA (siNK) and siRNA targeting ADHFE1 (siADHFE1). *n* = 6. Two-way ANOVA. (**e**) Per-cent of R- and S-2HG in MCF7 and Hs578T cells, Scr and IDH2^−/−^ clones. *n* > 6. Two-way ANOVA. * *p* < 0.05, *** *p* < 0.001, **** *p* < 0.0001, *ns* not statistically significant.

As IDH2 and ADHFE1 both produce 2HG in mitochondria directly, we hypothesized that both enzymes might compete for 2OG. To estimate ADHFE1 expression (alternatively hydroxyacid-oxoacid transhydrogenase, HOT, EC: 1.1.99.24), we quantified its mRNA levels in breast cancer cells (Figure 1b, Figure 3a). There is a marked difference in ADHFE1 expression among breast cancer cells, although overall expression levels of ADHFE1 are rather low compared to housekeeping genes *PPIA* and *ACTB* (Figure 1b, Figure 3a). According to Uniprot (Q8IWW8), there are 4 ADHFE1 protein isoforms expressed from a single mRNA variant by alternative splicing. The enzyme was characterized recently in terms of 2HG production and cancer biology [13], which delineated its clear role in 2HG production. 

To elaborate on the fact that ADHFE1 might be competing with IDH2 for 2OG, we used IDH2^−/−^ cells and compared the extent of 2HG production after the addition of artificial cell-permeable substrates dmOG and 3MP in MCF7 and Hs578T (Figure S3a,b). Elevated 2HG with dmOG in IDH2^−/−^ cells indicated the activity of 2HG production by enzymes other than IDH2 (Figure 3b—bottom blue bars, Figure S3a,b). The observed dmOG-stimulated 2HG production in Hs578T was abolished in IDH2^−/−^ cells by 30–50% in three independent experiments, while in MCF7 cells IDH2 contributed up to a 20% extent in dmOG-treated cells (Figure 3b, grey bars). That implies that sources other than IDH2 contribute to 2HG production more significantly in MCF7 than Hs578T. 

We have subsequently knockdowned ADHFE1 in MCF7 cells and performed the dmOG treatment in cells silenced for ADHFE1 to estimate the ADHFE1’s contribution. The silencing of ADHFE1 was confirmed by real-time PCR (Figure S3c). Strikingly, knockdown of ADHFE1 in MCF7 cells led to a compensatory increase of 2HG production (Figure 3c, blue box). The increase in 2HG after ADHFE1 silencing might be caused by a more efficient channeling of 2OG into the IDH2 reaction, or possibly the MDH2 reaction. We have therefore also silenced ADHFE1 in IDH2^−/−^ cells, which showed that dmOG-induced 2HG levels in ADHFE1-silenced cells were, indeed, IDH2-dependent (Figure 3c). 

We have also measured 2HG enantiomers to test whether the ratio of enantiomers may corroborate a source of 2HG in ADHFE1 knockdowned cells. Knockdown of ADHFE1 (48 h) led to a mild increase of R-HG, which suggests that 2OG was taken away by R-2HG producing reaction, possibly IDH2 (Figure 3d). On the other hand, IDH2 knockout results in a consistent increase of S-2HG, which suggests that 2OG in the absence of IDH2 might be channeled into the oxidative branch of the Krebs cycle, possibly MDH reaction (Figure 3e). To conclude, our data suggest there might be a vital competition for 2OG between 2HG producing reactions, including IDH2, ADHFE1, 2OGDH, and MDH2. The kinetic data regarding apparent affinities of the respective enzymes and local concentrations of the substrate would improve our understanding of the observed results.

### 2.4. Role of NADPH Balance in IDH2-Produced 2HG, Substrate, and Cofactor Flux

**Figure 4:**
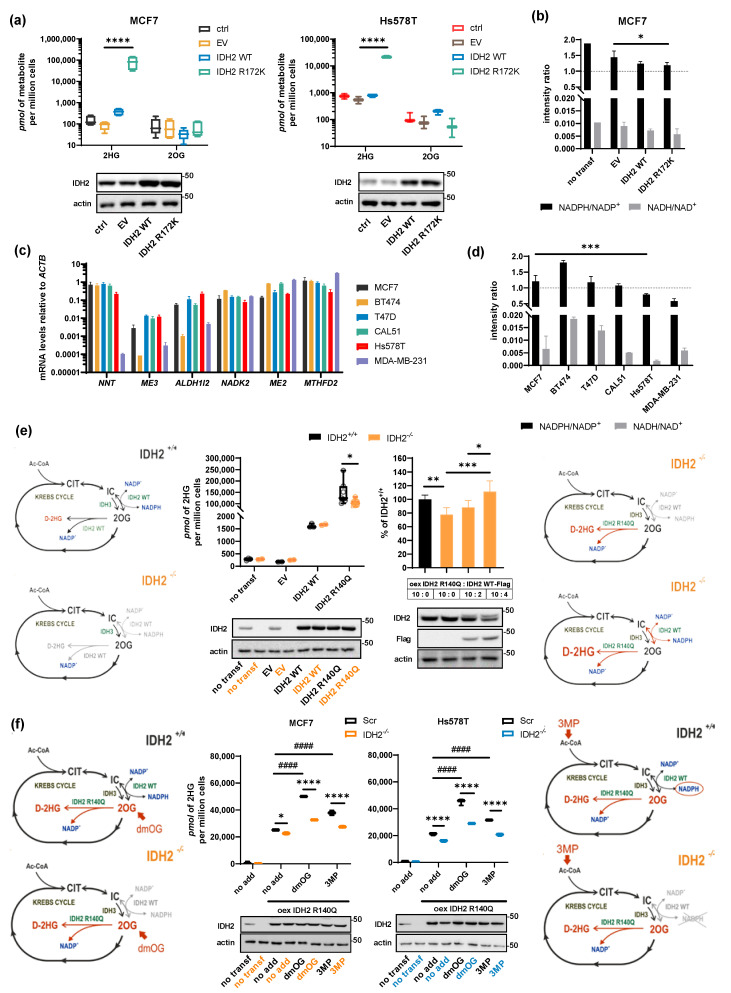
Cofactor balance regulates 2HG production. (**a**) Quantification of the expression level of genes related to mitochondrial NADPH production measured by qPCR or western blot. (**b**) The ratio of cofactors measured from whole-cell lysates in six breast cancer cell lines. *n* = 3. Two-way ANOVA. (**c**) 2HG and 2OG levels in MCF7 and Hs578T cells transfected with IDH2 WT and IDH2 R172K. *n* = 6 (MCF7), *n* = 3 (Hs578T). Two-way ANOVA. (**d**) The ratio of cofactors measured from whole-cell lysates in MCF7 cells transfected with IDH2 WT and IDH2 R172K. *n* = 3. Two-way ANOVA. (**e**) Middle-left, rescue effect of IDH2 R140Q in IDH2^+/+^ and IDH2^−/−^ cells, corresponding 2HG production. *n* > 3. Two-way ANOVA. Middle-right, expression of IDH2 R140Q and WT-Flag variant in 10:0, 10:2, and 10:4 ratios (µg: µg), respectively. *n* = 6. One-way ANOVA. Western blots depict IDH2 expression and IDH2-Flag expression (WT). Schemes depict the experimental design. (**f**) 2HG levels representing rescue effects provided by dmOG and 3MP treatment in Scr and IDH2^−/−^ cells expressing IDH2 R140Q. *n* = 3. Two-way ANOVA. Western blots depict IDH2 and actin expression; the order of lanes is identical to the boxes in the graphs above. Schemes depict respective experimental design. * *p* < 0.05 ** *p* < 0.01 *** *p* < 0.001 **** *p* < 0.0001, #### *p* < 0.0001.

We demonstrated that there is an uneven contribution of IDH2 and ADHFE1 to 2HG production in MCF7 and Hs578T cells, hence we might speculate that the NADPH balance is the factor contributing to the competition between IDH2 and ADHFE1. If we induce NADPH production in mitochondria, the IDH2 contribution would be elevated and ADHFE1-specific 2HG would be diminished. Thus, to further understand the requirements of NADPH in 2HG production in breast cancer cells, we overexpressed IDH2 mutant R172K, which produces excessive levels of 2HG while still requiring NADPH as a cofactor. Overexpression of IDH2 R172K increased 2HG in MCF7 cells, while moderately (by one order of magnitude less) increased 2HG in Hs578T cells (Figure 4a). That might suggest that the unbound matrix NADPH pool was not sufficient to support IDH2 reaction in Hs578T cells to the extent comparable to MCF7 despite relatively similar levels of IDH2 R172K after overexpression in both cell lines. It is intriguing, though, that mitochondrial metabolism can mobilize the NADPH pool sufficient to sustain 2HG production by IDH2 R172K. Indeed, overexpression of IDH2 R172K resulted in a significant decrease of the total cell NADPH/NADP^+^ ratio (Figure 4b).

We have analyzed the expression of plausible sources of NADPH production in mitochondria, namely NNT, ME2, ME3, MTHFD2, ALDH1l2, and mitochondrial NAD-kinase (Figure 4c, Figure S4a). The most abundant NADPH producer in the tested cell lines was usually MTHFD2, followed by NNT (Figure 4c). We have subsequently measured the total cell cofactors NADP^+^, NADPH, NAD^+^, and NADH by LC-MS and calculated the ratio of NADPH/NADP^+^, NADH/NAD^+^ for the six tested cell lines (Figure 4d). The observed NADPH/NADP^+^ ratio was higher in MCF7 compared to Hs578T cells (Figure 4d). Despite the estimated ratios probably reflect the situation in the whole-cell volume, we expect similar ratios in the mitochondrial matrix.

Additionally, in the oxidative mode (NADP^+^-dependent oxidative decarboxylation of IC into 2OG) IDH2 itself can be a donor of NADPH to the pool of enzymes performing the reduction of 2OG into 2HG, as demonstrated with purified IDH2 [9]. To provide evidence that IDH2 under certain conditions provides NADPH, we used IDH2^−/−^ MCF7 cells and overexpressed IDH2 WT or IDH2 R140Q (capable of neomorphic reduction of 2OG to 2HG, but not oxidative decarboxylation of IC or RC) to compare 2HG production at the background of IDH2^+/+^ and ^−/−^ cells, respectively (Figure 4e, left). Because intrinsic IDH2 is capable of 2OG and NADPH production, we expected that IDH2 R140Q overexpressed in MCF7-IDH2^−/−^ cells would provide lower 2HG levels in comparison to scrambled (IDH2^+/+^) cell line (Figure 4e). Indeed, MCF7-IDH2^−/−^ cells showed repeatedly lower levels of 2HG (on average by 33%), when transfected with IDH2 R140Q compared to scrambled cells (Figure 4e). The difference between R140Q transfected ^+/+^ and ^−/−^ cells should be attributed to intrinsic IDH2 output products, i.e., to the substrate or cofactor. To achieve a rescue effect of the observed difference, we further co-transfected IDH2^−/−^ cells (orange bars in Figure 4e, right) with IDH2 R140Q along with IDH2-WT-Flag variant (in 10:2 and 10:4 ratios, respectively). The transfection with IDH2 WT improved the IDH2 R140Q-related 2HG production in MCF7-IDH2^−/−^ cells up above a full compensation. That suggests that IDH2 WT, indeed, provides metabolic flux for the 2HG production by IDH2 R140Q. It is noteworthy that transfection of IDH2^−/−^ cells with EV:IDH2 WT in the same ratios (10:2, 10:4) results in much lower 2HG levels (Figure S4b). This suggests that IDH2 WT contributes to 2HG production by synergy and provides a substrate or cofactor to the pool of IDH2 molecules that actually produce 2HG.

To further examine if IDH2 WT supports R140Q preferably by substrate or cofactor, we treated IDH2 KO and scrambled cells by 3MP and dmOG, respectively. If the substrate (2OG) was the limiting factor between in ^−/−^ cells, dmOG should compensate the 2HG pool. However, treatment with dmOG does not induce 2HG to the same level as in IDH2^+/+^ cells, indicating that the substrate itself is not sufficient to provide optimum conditions for 2HG formation by IDH2 R140Q when IDH2 WT is lacking (Figure 4f). As stated above, 3MP treatment results in a mild yet consistent increase of 2OG but not 2HG levels (Figure 2e), indicating that 3MP entered the Krebs cycle in an oxidative direction. Therefore, we assumed that 3MP was utilized in oxidative decarboxylation by IDH3 and IDH2, producing 2OG, NADH, and NADPH, respectively. Similarly, to dmOG, 3MP treatment also induces 2HG more in IDH2^+/+^ cells than in IDH2^−/−^ cells (Figure 4f), indicating that oxidative substrate (3MP) in the absence of the intrinsic IDH2 cannot compensate for the substrate (2OG), nor cofactor (NADPH). However, we take notice that 3MP treatment results in mild induction of 2HG production, and a more potent oxidative substrate or reliable NADPH precursor would produce more conclusive data in this experiment. We therefore cannot exclude that cofactor NADPH is the main factor ensuring synergism between IDH2 molecules. We conclude that IDH2 WT can be a donor of substrate and cofactor by substrate channeling or providing bulk metabolic flux to mutated IDH2 as well as WT enzymes.

### 2.5. HG Is Released from Cells and Is Detectable in Urine and Plasma of Breast Cancer Patients

**Figure 5:**
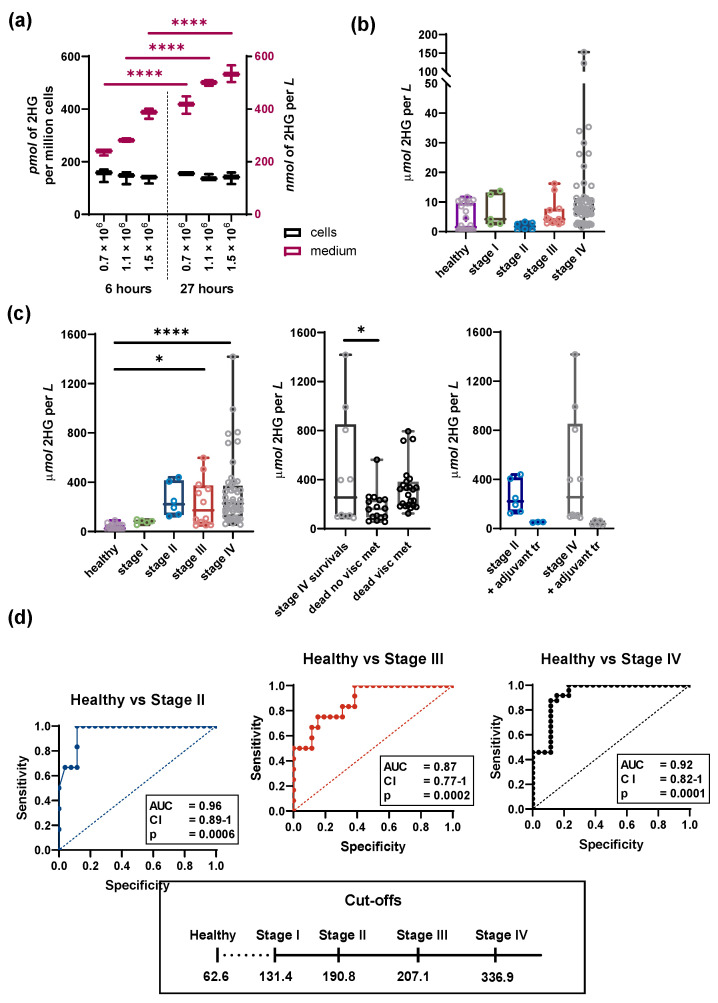
2HG release from cells and tumors. (**a**) 2HG levels released in medium (magenta) versus cell lysates (black) at the indicated cell density and time frame. (**b**) 2HG concentration in serum of the breast cancer patients and healthy volunteers. *n* > 5. (**c**) Left: 2HG concentrations in the urine of the breast cancer patients and healthy volunteers. *n* > 5. One-way ANOVA. Middle: Urine concentrations of the patients in stage IV; including with or without visceral metastases (*visc met*). Right: Urine concentrations of the patients in stages II and IV, respectively, indicating the second estimation after 1 or 6 months of anti-HER2 treatment with Trastuzumab and chemotherapy. (**d**) ROC curves of the urine 2HG levels and calculated cut-off values. * *p* < 0.05 **** *p* < 0.0001.

There is an intriguing question regarding the relevance of 2HG release into the bloodstream and excretion into the urine in a surplus above the normal metabolism. We asked what the relationship between intracellular and extracellular concentrations of 2HG is. Using cultured cells, we quantified 2HG concentrations in cell lysates vs. culturing medium of three different densities of the cell population and in the medium replaced 6 or 27 h prior to analysis, respectively. The experiment should have excluded the possibility that 2HG accumulated in the cells with prolonged culturing or clonal development, and to verify that the intracellular concentration of 2HG reflects the actual intracellular homeostasis of 2HG-producing vs. consuming factors. Nevertheless, the extracellular concentrations of 2HG might depend on the elevated tumor or metastasis mass. The data demonstrate that the intracellular 2HG concentration remains constant regardless of the extracellular concentration of 2HG and that 2HG is exported from the cells at a constant rate (Figure 5a). On the other hand, the intracellular concentration of 2HG is relevant for the analysis of 2HG production pathways and very reproductive.

We also performed a clinical study, analyzing urine and serum total 2HG levels (R-2-HG plus S-2-HG) in 28 breast cancer patients after resection of their tumors (Table S2). We aimed to analyze, whether cancer-specific metabolism results in the accumulation of 2HG, and whether 2HG in urine or serum could serve as a diagnostic marker of breast carcinoma. Correlations were found between the urine 2HG levels and patients’ stage, amount, and character of visceral metastases and mortality (Figure 5c). The calculated correlation (R^2^ ~ 0.3; neglecting stage IV patients with only bone metastases) fits a range from zero to 400 μmol 2HG per liter, whereas levels of 400 to 1400 μmol per liter were found in ~25% of patients. Some of these correlations were stronger, e.g., when 2HG was normalized to creatinine, 2OG, or citrate (Table S1, Figure S6) while being less pronounced concerning the plasma 2HG levels (Figure S6b). The median of urine 2HG concentration for the studied group of 28 patients was 300 μmol per liter (maximum 1120 μmol per liter). Very low 2HG background levels (4–34 μmol per liter) were found in the urine of healthy volunteers (Figure 5c), whereas their plasma levels were slightly higher on average (Figure 5b). In three cases, we found lowered 2HG levels during anti-HER2/chemotherapy treatment (Figure 5c).

Receiver operating characteristic curves (ROC) supported the idea of using 2HG urine levels as a metabolic marker of metastasis progression and/or breast cancer recurrence, while urine 2HG levels and 2HG/citrate ratios could be distributed to ranges corresponding to distinct stages and, most importantly, indicated visceral metastases or predicted mortality (Figure 5d, Figure S5). 2HG levels by themselves clearly distinguished healthy patients (Figure 5d). ROC analyses showed a distinction between patients with visceral metastases from those with only bone metastases.

## 3. Discussion

In this work, we addressed the question of mitochondrial substrate flux and its role in the production of 2HG in breast cancer cells. In general, we emphasize the role of mitochondrial enzymes ADHFE1 and IDH2 in the synthesis of oncometabolite 2HG. We demonstrated that IDH2 and ADHFE1 activities are mutually interrelated, affecting each other in extent depending on 2OG and possibly also NADPH availability. Furthermore, we also revealed the clinical relevance of 2HG detection in the urine of breast cancer patients.

Our findings are based on the measuring of 2HG levels in breast cancer cultured cells. In the tested cell lines, there is a considerable production of 2HG, which is significantly decreased by knockout of IDH2 and silencing of ADHFE1. Here we show that the R-2HG enantiomer represents up to 70% of the total 2HG pool in tested cell lines. It has been established formerly that the glutaminolysis flux is the principal carbon source in reductive reactions derived from the Krebs cycle [24,25]. In this study, we demonstrate that promoting or inhibiting glutaminolysis regulates 2HG production positively and negatively, respectively. Our data suggest that there might be a variable degree of 2OG saturation, hence manipulation with glutaminolysis can induce various degrees of changes in 2HG production in the tested cell lines based on the actual 2HG-producing capacity. Interestingly, we show that under steady-state conditions, 2HG levels in MCF7 are limited by 2HG-producing capacity, while in Hs578T rather by the 2OG supply. Importantly, other enzymes compromise glutaminolysis substrate flux, including mitochondrial branched-chain aminotransferase (BCAT2), glutamine synthase (GS, *GLUL*), or xCT transporter (glutamate-cysteine antiporter, *SLC7A11*), activities of which could result in a decline of 2OG levels available for the Krebs cycle.

So, we asked whether there is a causal relationship between the extent of IDH2 and the ADHFE1-related 2HG production. The extent of 2HG production by ADHFE1 is negatively regulated by the IDH2 activity which is, in turn, regulated by the NADPH availability. Hence, we hypothesized that the NADPH availability benefits IDH2 reaction in cells with the elevated NADPH/NADP^+^ ratio, i.e., MCF7 over Hs578T cells. On the contrary, ADHFE1 more actively contributes to 2HG synthesis in MCF7 cells than in Hs578T cells. Moreover, we show that inhibition of ADHFE1 benefits IDH2 reaction in MCF7 cells (Figure 3c,d). Mechanistically, such competition might depend on the apparent affinities and local substrate concentrations in the mitochondrial matrix, which is vastly compartmentalized by the cristae space. However, we were not able to demonstrate specific ADHFE1 and IDH2-related fluxes by genetic in combination with biochemical methods, because stable isotope tracing cannot distinguish 2HG produced in the ADHFE1 or IDH2-specific reactions. Likewise, the catalytical role of ADHFE1 is still rather unexplored; ADHFE1 catalyzes the reactions of 3-hydroxybutanoate or 4-hydroxybutanoate into acetoacetate or succinate semialdehyde, respectively, coupled to 2OG reduction into 2HG. Consequently, in-depth analysis of metabolic fluxes in the mitochondrial matrix would be helpful to estimate actual 2HG producing capacities.

Subsequently, we are also intrigued by the consideration that wild-type IDH2, running in the oxidative decarboxylation mode of IC into 2OG, might be an NADPH source itself for a specific fraction of IDH2 molecules generating 2HG. We, therefore, addressed the question of synergism between IDH2 molecules in mitochondria. We acknowledge that IDH2 WT is indeed dispensable for 2HG production by mutated IDH2 (R140Q), but when present, contributes to 2HG production by substrate or cofactor provision (Figure 4). Rescue effects were provided by overexpression of IDH2 WT, which compensated for the loss of 2HG. Our data suggest that intrinsic IDH2 WT provides bulk or local metabolic flux towards reduction by mutated IDH2, and possibly also wild-type IDH2. However, our data are in contrast with the work of Ward et al. [26] who did not observe a difference in 2HG production catalyzed by IDH2 R140Q or R172K at the background of cells expressing intrinsic IDH2 vs. silenced intrinsic IDH2 or IDH3, respectively. Our study also partially overlaps with Dexter et al. [27] who addressed the issue of retention of wild-type variant of IDH1 and rejected the concept of synergy within IDH1 heterodimers due to the short distances between active sites within the dimer. The addition of permeable mitochondrial substrate analogs dmOG and 3MP did not fully recover 2HG production in IDH2^−/−^ cells. So, our assay failed to demonstrate if 2OG or NADPH, respectively, is actually channeled within IDH2 pools or fractions. However, an inducer of mitochondrial NADPH or permeable form of NADPH or its precursor would be helpful to perform the relevant experiment. We conclude that channeling of substrate/cofactor is possible between mitochondrial IDH2 molecules working in oxidative/reductive mode in synergy, respectively. It is exciting to consider similar cooperation within IDH2 WT/IDH2 WT dimer.

We also tested if 2HG is maintained in vivo in the bloodstream and urine of breast cancer patients and whether such analysis can be used as a diagnostic or prognostic marker. It was not known, whether, in typical breast carcinoma cases, the cancer-specific metabolism promotes the formation of oncometabolite 2HG and build-up of 2HG in the circulation. We found significantly elevated urine 2HG levels (but not serum) in breast cancer patients undergoing adjuvant therapy. The lowest 2HG levels in urine were found in healthy volunteers. Previously, substantial 2HG levels were found in the patient’s body fluids such as in urine, plasma, or cerebrospinal fluid [7,11], in some cases ascribed to the existing oncogene changes [11,13]. Despite studying a heterogenic group of 28 breast cancer patients (Table S2), such simple diagnostics seems to be promising. Importantly, the study yielded positive ROC analyses and cut-off values (Figure 5, Table S1) and exhibited a span of up to three orders of magnitude for urine 2HG levels from healthy volunteers up to the highest levels found in some stage IV patients. Our study aimed to reveal potential correlations between 2HG levels in urine/serum and cancer stage. Hypothetically, 2HG levels above a certain threshold can indicate the remaining presence of metastases even after tumor resection. Nevertheless, it is necessary to verify the fidelity of these possible predictions in larger clinical studies. From the clinical perspective, it would be useful to focus on the verification of 2HG as a possible marker of early breast cancer, because such markers are completely missing in the clinical practice. Moreover, it will be also necessary to crosscheck blood and urine 2HG levels with expression and genotype of IDH2, ADHFE1, and other 2HG-producing enzymes in the biopsy samples. Future screening of both neoadjuvant and adjuvant patients of various disease stages is required to validate our study and to demonstrate that 2HG levels may also indicate the early onset of disease or minimal residual disease.

## 4. Materials and Methods

### 4.1. Cell Cultures

In the study, we used commonly used commercial human breast cancer cell lines. BT474 and T47D were maintained in RPMI (BT474, T47D) or DMEM (MCF7, Hs578T, CAL51, MDA-MB-231) containing 10% FBS, 1.5 g·L^−1^ of NaHCO_3_, 10 mM HEPES, 1× penicillin/streptomycin, 1 mM sodium pyruvate, 5 mM glucose, and 4 mM glutamine. Cell lines expressing estrogen receptor (MCF7, BT474, T47D) were maintained in phenol-red-free media supplemented with 10 nM 17-β-estradiol. Transfection of vector DNA was performed using Lipofectamine2000 (Thermofisher Scientific (Waltham, MA, USA)) or GeneGet transfection reagent (SignaGene) according to the manufacturer´s protocols. siRNA was transfected using RNAiMAX transfection reagent (Thermofisher Scientific). Knockdown of ADHFE1 was performed using a mixture of three predesigned Stealth siRNAs purchased from ThermoFisher Scientific (HSS134689, HSS13469, and HSS175147). IDH2 WT fused with a Flag-tag was a kind gift from the laboratory of Dr. Denu [28]. Vectors encoding IDH2 R140Q, IDH2 WT, and IDH2 R172K were kind gifts of Craig Thompson´s Laboratory. Vector encoding GAC K320A was a kind gift of Dr. Sandra Martha Gomes Dias [23].

### 4.2. Crispr-Cas9

Deletion of IDH2 in MCF7 and Hs578T cells was performed using CrisprCas9 technology. gRNA was designed using CRISPOR, a web-based tool for genome editing experiments with the CRISPR–Cas9 system (http://crispor.tefor.net accessed on 1 May 2020). sgRNA sequence (5′GGCCACCCAGAAGTACAGTG) was subcloned into LentiCRISPR (pXPR_001) vector (Addgene) and transfected into MCF7/Hs578T cells. Cells were treated with 1 µg/mL puromycin for 10 days and then cultured for three weeks to generate a pool of IDH2 deleted cells. IDH2 deletion was verified by western blot. Nonsense sgRNA sequence (5′CGCACTACCAGAGCTAACTCAGATAGTACT) was used in parallel as a negative control (scrambled, Scr).

### 4.3. Analytical Techniques

Cofactor measurement followed the protocol described in Lu et al. [29]. Briefly, cell pellets were washed with 137 mM NaCl and 2.7 mM KCl and subsequently resuspended in a buffer containing 40% acetonitrile, 40% methanol, 20% water, and 0.1 M formic acid, neutralized with 15% NH_4_HCO_3_ (*w*/*v*) after 3 min and incubated for 20 min on ice. Samples were centrifuged at 16,000× *g* for 15 min and the supernatants were analyzed by LC-MS analysis. The analysis was performed in the BIOCEV OMICS facility. The samples were analyzed on Dionex Ultimate 3000RS liquid chromatography system coupled to a TSQ Quantiva mass spectrometer (ThermoScientific) using ESI source in negative mode with the following ion source parameters: ion transfer tube temperature 325 °C, vaporizer temperature 200 °C, spray voltage 2500 V, sheath gas 35 and aux gas 5. A ZIC^®^-pHILIC column (150 mm × 2.1 mm, 5 µm) from Merck (Darmstadt, Germany) was used for the separation of analytes. The column was maintained at room temperature with an injection of 1 µL of the sample. The gradient elution was set from 15% A to 75% A (A: 10 mM ammonium carbonate in water, pH 9.3; B: 97% acetonitrile) in 11.5 min at the flow rate of 130 µL/min, followed by a washing phase (5 min of 75% A flow) and equilibration phase (10 min of 15% A flow). For targeted determination of analytes, a selective reaction monitoring (SRM) mode was developed previously by infusing pure compounds.

For GC-MS measurement of cell metabolites 2HG, 2OG, citrate, malate, and fumarate cells were transfected and/or treated as desired. Subsequently, cells were harvested, washed with PBS, counted, and frozen until analyzed. The analysis was performed as described previously [9].

For metabolomics of polar metabolites, cells were grown on a 6-well plate, washed with PBS, and frozen. LC-MS-based metabolomics profiling was performed at the Department of Metabolomics, IPHYS CAS. Metabolites were extracted using a biphasic solvent system of cold methanol, methyl tert-butyl ether, and water [30]. An aliquot of the bottom (polar) phase was collected, cleaned up using an acetonitrile/isopropanol mixture, and after evaporation, the dry extract was resuspended in 5% methanol with 0.2% formic acid followed by separation on an Acquity UPLC HSS T3 column (Waters). Another aliquot of the bottom phase was evaporated, resuspended in an acetonitrile/water mixture, and separated on an Acquity UPLC BEH Amide column. Metabolites were detected in negative and positive electrospray ion mode (Thermo Q Exactive Plus instrumentation), respectively [31]. Signal intensities were PQN normalized before subsequent statistical analysis.

For chiral separation of 2HG enantiomers, we used the protocol described in Cheng et al. [32]. Briefly, cell samples were extracted with water/methanol/chloroform (1:1:2, *v*/*v*/*v*) and centrifuged at 3000× *g* for 10 min. The polar water/methanol phase was collected into a glass vial and lyophilized overnight. R/S-2-HG were then labeled with N-(p-toluenesulfonyl)-L-phenylalanyl chloride (Santa Cruz Biotechnology, CA, USA) having concentration 2.5 mmol/L using anhydrous acetonitrile as a solvent. Derivatization was performed at room temperature for 15 min. The mixture was dried with nitrogen gas and redissolved in 100 µL of 50% aqueous acetonitrile. Chiral analytes were separated with HPLC system (Dionex Ultimate 3000, Dionex Softron GmbH) equipped with Inertsil ODS-3 (150 mm × 2.1 mm, 5 µm, GL Sciences). A binary mobile phase system was used consisting of water with 0.1% formic acid (A) and methanol/acetonitrile 1/1 (B) starting 70/30 (A/B), the flow rate was set at 0.2 mL/min. R-2HG and S-2HG were detected in a triple quadrupole mass spectrometer (TSQ Quantum Access Max with HESI-II probe, Thermo Fisher Scientific) operating in a negative mode (the mass transitions for labeled analytes were 447.9–150.0).

### 4.4. Chemicals

We used the following specific chemical compounds for cell treatment: (aminooxy)acetic acid hemihydrochloride (Sigma-Aldrich C13408), CB-839 (Focus Biomolecules 10-4556), BPTES (Sigma-Aldrich, SML0601), dmOG (Sigma-Aldrich, 349631), methyl pyruvate (Sigma-Aldrich, 371173), 17-β-estradiol (Sigma-Aldrich, E2758), octyl-R-2HG (Sigma-Aldrich, SML2200). Antibodies used in the study were following: Gls1 (Abcam ab156876), PHGDH (Abcam ab57030), anti-FLAG (M2, Sigma-Aldrich F1804), IDH2 (Abnova H00003418-M01), ATP5A (Abcam ab14748), Tim23 (Abcam ab176558), PDH (Abcam ab92696), and β-actin (Abcam ab8226).

### 4.5. Real-Time PCR

Real-time PCR was performed using a LightCycler480 instrument (Roche) or CFX Connect Real-Time PCR System (BioRad), respectively. RNA was isolated using Total RNA Purification Kit (Jena Bioscience), followed by reverse transcription using TATAA GrandScript cDNA Synthesis Kit (TATAA Biocenter). Q-PCR was performed using Forget-Me-Not EvaGreen qPCR Master Mix (Biotium). Calculated crossing points were used to calculate ΔCt values. Z-scores were calculated from respective ΔCt values. Primer sequences are summarized in Table S3.

### 4.6. Clinical Study

The study was approved by the Ethics Committee of the General University Hospital, Prague, clinical study No. 48/17 (The General University hospital and the First Faculty of Medicine). Female Caucasian individuals (*n* = 28) with metastasizing breast cancer staged according to the TNM system were studied post-operation, when metastases were verified by routine imaging methods. The adjuvant treatment included chemotherapy, hormonal or HER2-targeted therapy. Patients were grouped as described in Table S2. Samples of serum (7 mL) and urine (100 mL) were carried during the routine follow-up and stored in −80 °C prior to analysis. The clinical-pathological characterization of the examined subjects included histological type, tumor grade, and determination of hormonal status (estrogen, progesterone receptors), overexpression of HER2neu, and BRCA mutation status. Urine and plasma samples were frozen and analyzed using the GC-MS instrument, as described above. GraphPad Prism 8.0 was used for the calculation of the receiver operating characteristic curves (ROC), deriving sensitivity, specificity, and cut-off values.

### 4.7. Statistical Analysis

All data are expressed as means and standard deviations (SD). Whisker plots are depicted from minimum to maximum values. Statistical significance was calculated using GraphPad Prism 8 software using one-way or two-way analysis of variance (ANOVA) followed by multiple comparison testing (Tukey’s, Dunnett’s, and Sidak’s, recommended by software). When relevant, a t-test was performed. Statistically significant *p*-values are presented in the figures; * *p* < 0.05, ** *p* < 0.01, *** *p* < 0.001, **** *p* < 0.0001. For volcano plots, *p*-values were calculated using RStudio, version 1.3.1073, using the built-in function *t*-test.

## 5. Conclusions

We demonstrate a spectrum of 2HG producing phenotypes in breast cancer cell lines. We clearly distinguish the IDH2-dependent and independent production of 2HG in mitochondria and demonstrate the active competition between the 2HG producing machinery in mitochondria, namely ADHFE1 and IDH2 in breast cancer cells in vitro. Moreover, we provide evidence for synergistic substrate channeling between fractions of IDH2 molecules. The importance of 2HG by breast cancer cells can be emphasized by the fact that 2HG can be readily detected in breast cancer patients even after tumor resection and might be used as a prospective non-invasive marker of breast carcinoma and progression of metastases. It is yet unclear whether at all these levels reflect ongoing carcinogenesis, including metastases formation. In summary, we provide an experimental background for the substrate dependence in mitochondrial reactions leading to the production of the oncometabolite 2HG with possible implications for cancer biology.

## Supplementary Materials

The following are available online at https://www.mdpi.com/article/10.3390/cancers13071709/s1. Figure S1: Supplemental graphs to Figure 1. Figure S2: Supplemental graphs to Figure 2. Figure S3: Supplemental graphs to Figure 3. Figure S4: Supplemental graphs to Figure. Table S1: Cut-off values derived from the corresponding ROC curves. Table S2: Stratification of patients. Table S3: List of primer sequences used for qPCR analysis in the study. Figure S5: Supplemental graphs to Figure 5. Figure S6: Creatinine values in the urine samples of breast carcinoma patients. Figure S7: Full-length western blots presented in the manuscript.
