# Supplementary material for: Biochemical Background in Mitochondria Affects 2HG Production by IDH2 and ADHFE1 in Breast Carcinoma
Source: Cancers (Basel). 2021 Apr 4;13(7):1709. doi: 10.3390/cancers13071709 (PMC8038481; doi:10.3390/cancers13071709)
Supplement: Supplementary file 1 [file cancers-13-01709-s001.pdf]

cancers

MDPI

Supplementary information

Biochemical background in mitochondria affects 2HG production by IDH2 and ADHFE1 in breast carcinoma

Jitka Špačková[1], Klára Gotvaldová[1], Aleš Dvořák[1], Alexandra Urbančoková[1], Kateřina Pospíšilová[2], David Větvička[3], Alberto Andrés Leguina-Ruzzi[1], Petra Tesařová[4], Libor Vítek[2, 5], Petr Ježek[1] and Katarína Smolková[1,*]

[1] Institute of Physiology of the Czech Academy of Sciences, Laboratory of Mitochondrial Physiology, Prague, The Czech Republic; jitka.spackova@fgu.cas.cz, klara.gotvaldova@fgu.cas.cz, alesh.dvorak@gmail.com, alexandra.urbancokova@fgu.cas.cz, AlbertoAndres.LeguinaRuzzi@fgu.cas.cz, petr.jezek@fgu.cas.cz

[2] Institute of Medical Biochemistry and Laboratory Diagnostics of the General University Hospital and The First Faculty of Medicine, Charles University, Prague, The Czech Republic; pospisilova.kp@gmail.com

[3] Institute of Biophysics and Informatics, The First Faculty of Medicine, Charles University, Prague, The Czech Republic; david.vetvicka@gmail.com

[4] Department of Oncology, The General University Hospital and The First Faculty of Medicine, The Czech Republic; tesarova.petra@seznam.cz

[5] 4th Department of Internal Medicine, The General University Hospital and The First Faculty of Medicine, Charles University, Prague, The Czech Republic; vitek@cesnet.cz

* Correspondence: katarina.smolkova@fgu.cas.cz; Tel.: +420-296-442-285, IPHYS CAS, Vídeňská 1083, 14220 Prague 4 – Krč, The Czech Republic

cancers

MDPI

Figure S1

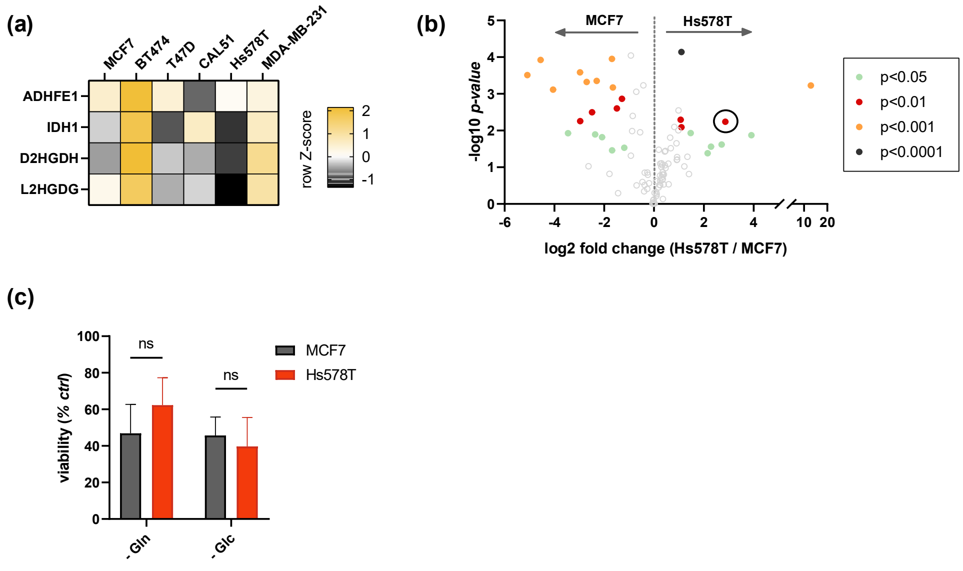

Figure S1. Supplemental graphs to Figure 1. **(a)** Heat-map of Z-score values for quantification of expression level of genes related to 2HG production measured by qPCR. **(b)** Volcano plot of polar metabolites derived from additional metabolomics experiment. 2HG is indicated in black circle. **(c)** Cell viability of MCF7 and Hs578T in glucose-free and glutamine-free media measured by neutral red. N > 5. One-way ANOVA. Cell viability of MCF7 and Hs578T in glucose-free and glutamine-free media measured by neutral red. N > 5. One-way ANOVA.

Figure S2

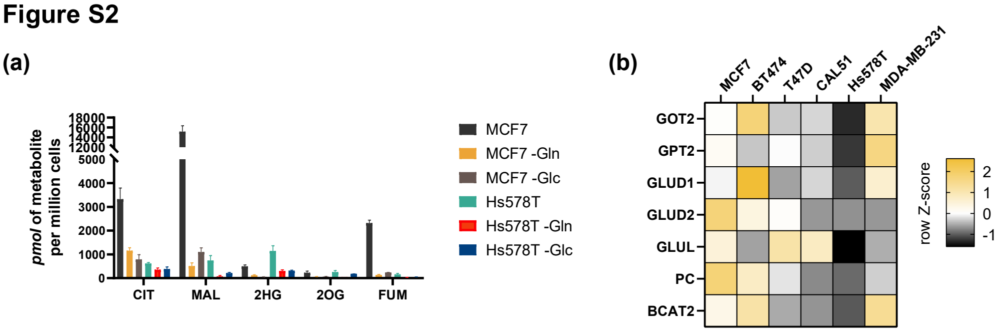

Figure S2. Supplemental graphs to Figure 2. **(a)** Metabolites of TCA cycle affected by removal of glutamine and glucose. **(b)** Heat-map of Z-score values for quantification of expression level of genes related to mitochondrial handling of glutamine measured by qPCR.

Figure S3

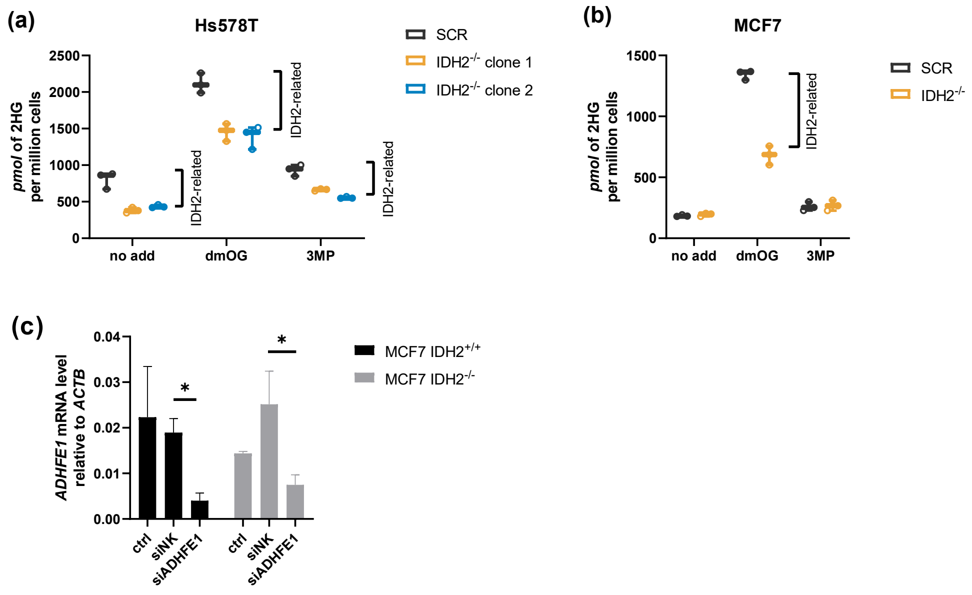

Figure S3. Supplemental graphs to Figure 3. The respective experiments were used for calculation of IDH2 related and unrelated 2HG production (Figure 3b) **(a)** 2HG production in Hs578T; non-treated cells, scrambled controls (SCR), and two populations of IDH2 KO cells. **(b)** 2HG production in MCF7 cells; non-treated cells, scrambled controls (SCR) and IDH2 KO cells. **(c)** Gene expression changes of ADHFE1 demonstrating knockdown of ADHFE1 in the experiment depicted in the Figure 3c.

cancers MDPI

Figure S4

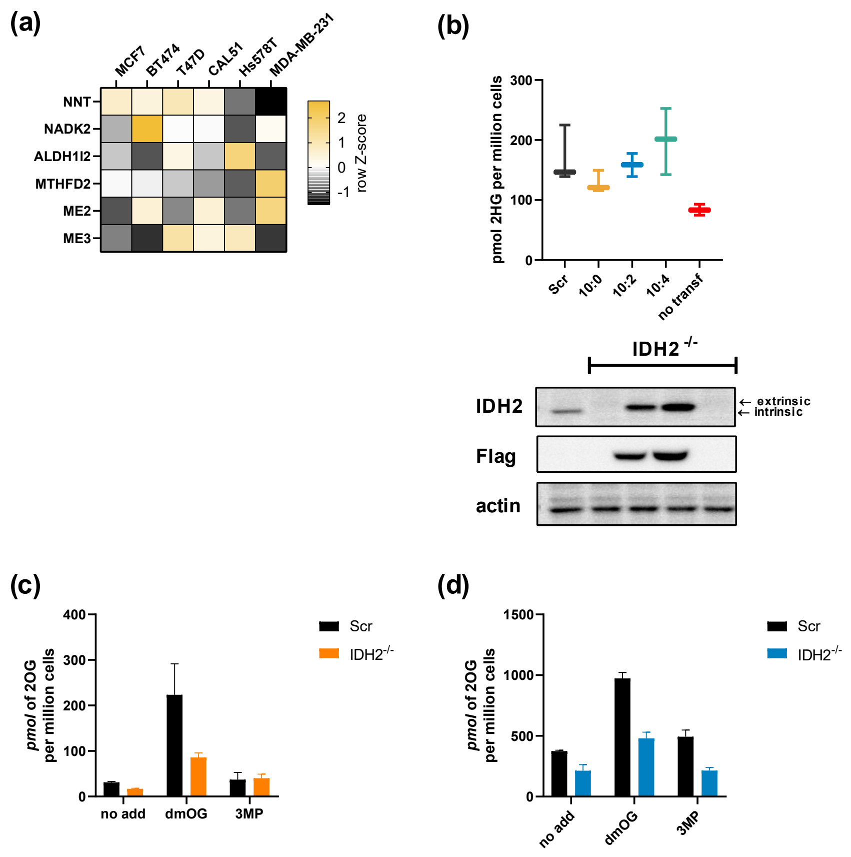

Figure S4. Supplemental graphs to Figure 4. **(a)** Heat-map of Z-score values for quantification of expression level of genes related to mitochondrial NADPH production measured by qPCR. **(b)** Production of 2HG: control experiment to Figure 4d; overexpression of IDH2 WT in IDH2 KO (MCF7) cells without co-overexpression of IDH2 R140Q. Below are western blots of the respective experiment. Lanes correspond to boxes in the graph above. **(c) (d)** 2OG production, experiment related to Figure 4F. 2OG production in MCF7 (SCR and IDH2 KO, respectively), overexpressing IDH2 R140Q treated with dmOG and 3MP. Right: same, in Hs578T SCR and KO cells.

cancers

MDPI

Figure S5

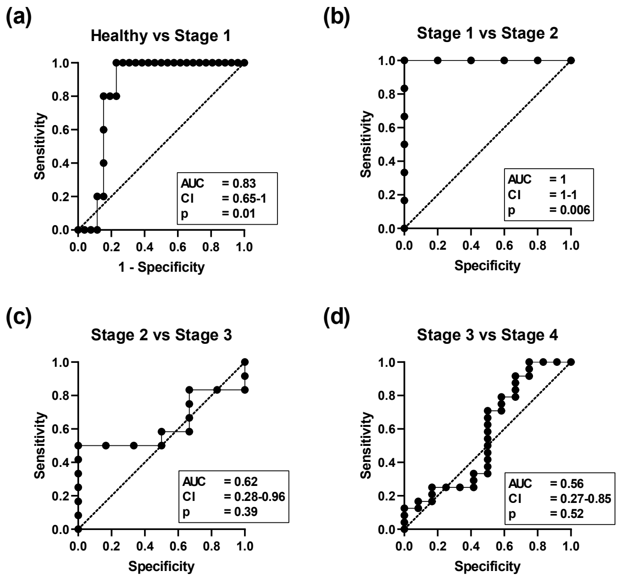

Figure S5. Supplemental graphs to Figure 5. **(a) (b) (c) (d)** ROC curves analysis of urine 2HG absolute levels between the indicated combinations of original patient's stages and derived areas under the curve (AUC), 99% confidence intervals (CI) and p values.

Figure S6

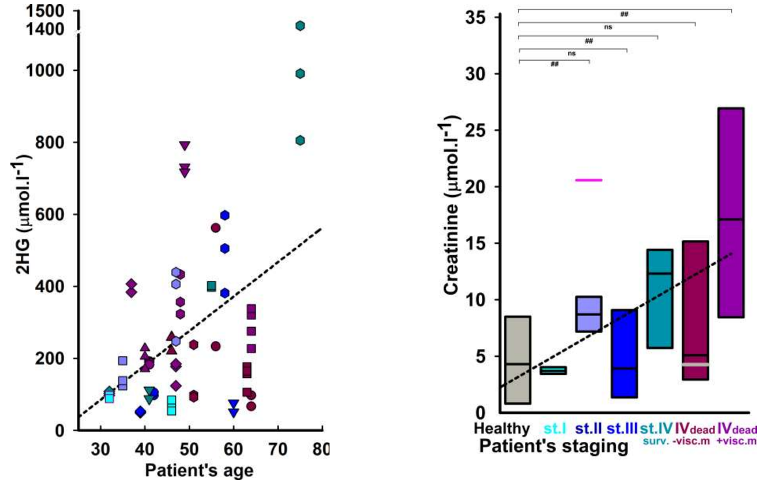

Figure S6. Creatinine values in the urine samples of breast carcinoma patients. **(a)** Creatinine in and patient´s age. **(b)** Creatinine stratified by patients' stages.

cancers MDPI

Table S1. Cut-off values derived from the corresponding ROC curves. Cut-off values for the absolute 2HG levels (in µmol ·l^{-1}), or normalized to creatinine, 2OG, and citrate, respectively, between the major stage groups of patients.

Normalized to:	**Healthy**	cutoff	**Stage I**	cutoff	**Stage II**	cutoff	**Stage III**	cutoff	**Stage IV**
**2HG**		70		124		345		402	
**Creatinine**		18		26.8		41		87	
**2OG**		1.5		2		3.6		5.8	
**Citrate**		0.23		0.3		0.47		0.64	

cancers

MDPI

Table S2. Stratification of patients. Summary of the treatment used in this study and urine 2HG levels ($\mu mol \cdot l^{-1}$) obtained by averages of average values for each patient. N = 28. 3- stands for the negativity of expression markers ER (estrogen receptor), PR (progesterone receptor), and HER2.

Disease info			Tretment / treatment details		Markers	Urine 2HG (nmol . L^{-1})		
Healthy			No treatment		N/A	33 ± 27		
Stage I	Remission	Localized	Adjuvant	ChemT	3-; BRCA+	82 ± 11	203 ± 152	
Stage II	Remission	Advanced	Adjuvant	ChemT + Trastuzumab	3-; ER+	189 ± 130		
Stage III	Remission /<br>Recurrence	Locally advanced	Adjuvant	No; Horm T<br>No; Chem T; Horm T	3-; ER+<br>3-; 3-; BRCA+	202 ± 172		
Stage IV	Surviving	Visceral metastases (not in 1 case)	Adjuvant/Palliative	Chem T + Horm T<br>Trastuzumab + Pertuzumab<br>Trastuzumab + Pertuzumab + Denosumab	BRCA+ ER+;<br>HER2+ ER+;<br>HER2+	201 ± 140		315 ± 256
	Dead	Only bone metastases	Adjuvant/Palliative	Trastuzumab; No; Chem T<br>Trastu + Pertu; Horm T	HER2+; HER2+ ER+; same<br>HER2+ ER+; HER2+ ER+	156 ± 59	280 ± 175	
	Dead	Visceral metastases / terminal phase	Adjuvant/Palliative	No; Chem T; Chem T; Chem T<br>Horm T; Chem T + Trastu; Kadcyla	3-; 3-; BRCA+, ER+;<br>ER+; HER2+ ER+; HER2+ ER+	335 ± 191		

cancers MDPI

Table S3. List of primer sequences used for qPCR analysis in the study.

gene	*forward primer 5´>3´*	*reverse primer 5´>3´*
hADHFE1	ATGGCCTTTCTGTGGTGCTC	GGTGTCGGCTCCCAGTATTT
hD2HGDH	TCCCCGTCTTTGACGAGATCA	AAGTCCCGTTCCTCCACATAC
hL2HGDH	CTGTTCACCAGACTGGACATAAC	GCCTTTCTCATATAGGGCCTGA
hIDH1	GGGAGTCCGGTTTGGGATTG	TCTCTACCACAGAACCGCCA
hGOT2	CCATGTATTCCAACCCTCCCC	GAGTCCGCATGCCAATGATG
hGPT2	CCCATCCCACAATATCCCCTC	GTTCCCAGGGTTGATTATGCAG
hGLUD1	TCCAGCAGACATTAAAAGCCC	CAGCCAGACTCCAAACAGGG
hGLUD2	TCAATCCCAAGAACTATACCGAAA	ATAGTGCCCTATGGTGCTGG
hGLUL	TCATCTTGCATCGTGTGTGTG	CTTCAGACCATTCTCCTCCCG
hPC	ACAGAGGTGAGATTGCCATCC	CACTGCATCTACGTTGTTCTCC
hBCAT2	GCTCAACATGGACCGGATG	CCGCACATAGAGGCTGGTG
hNNT	GGGGTCCTGTAAGGGTCTAC	ATGCCACTCGCTTCTCATTTT
hME3	TGAAGAAGCGCGGATACGATG	GAAAGCAGGGCGGGATTAGG
hALDH1l2	CTCCACTGGCCGGGTTTATT	AGCCAGAGGGTCAGCTTTTC
hNADK2	GCCTGCCCGTTCGATATACA	GTGCTCTCACTGGCAGAAGT
hME2	ATATACACCGACGGTTGGTCT	CATCAGTCACTACAACAGCCTT
hMTHFD	TACTCCATGGGGTGTGTGGG	ATATTGTAACAGTGGCATCACCTCC
hACTB	GAGCACAGAGCCTCGCCTT	GTGTAGAGCCGCAGAAGCAG
hPPIA	GTATAAAAGGGGCGGGAGGC	CTGCAAACAGCTCAAAGGAGAC
hADHFE1	TGCCTATGTTGCTGTCGGTG	TCCCGGTTCCTGAGGTAGTT
hADHFE1	TTGGCAAGTGCTTTTGCTGG	CATCTCCAGGTGTCGCTCTG

cancers

MDPI

Figure S7

(a)

Figure 1 a

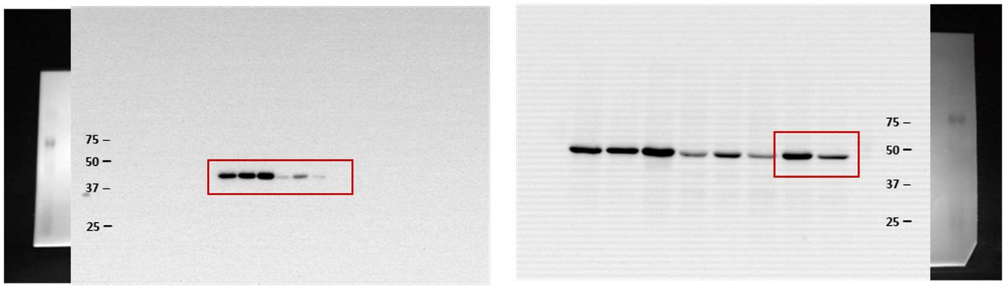

Figure 1 d

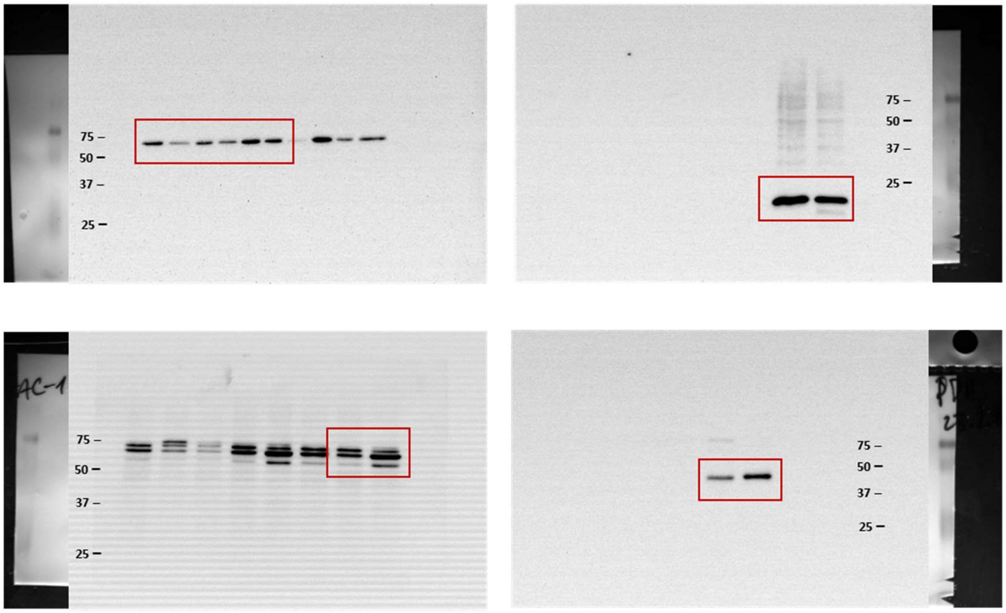

cancers MDPI

(b)

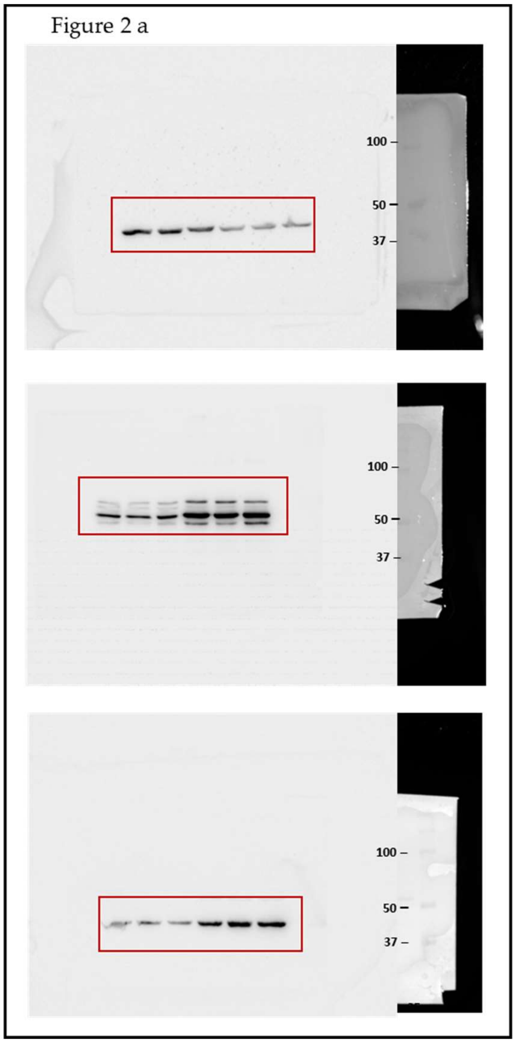

(c)

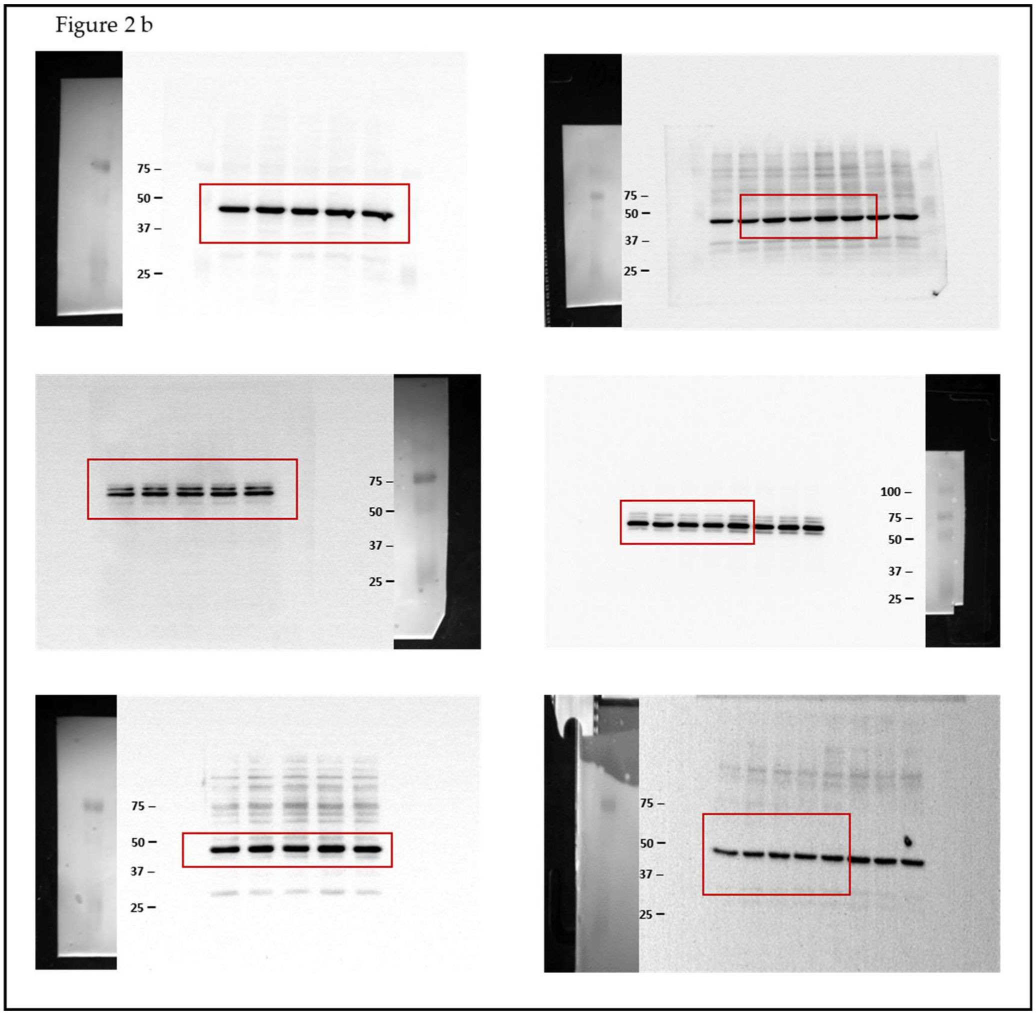

(d)

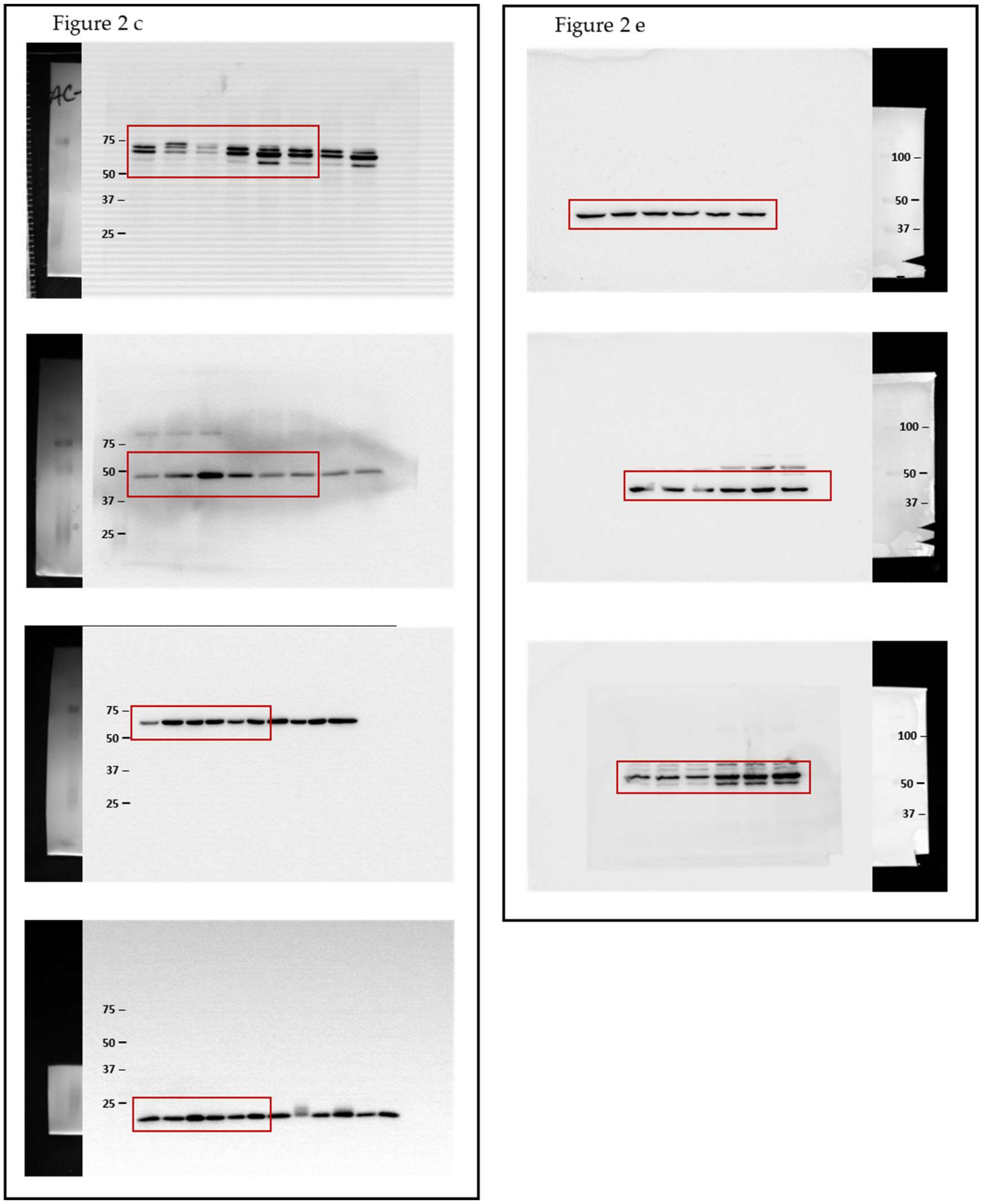

cancers

(e)

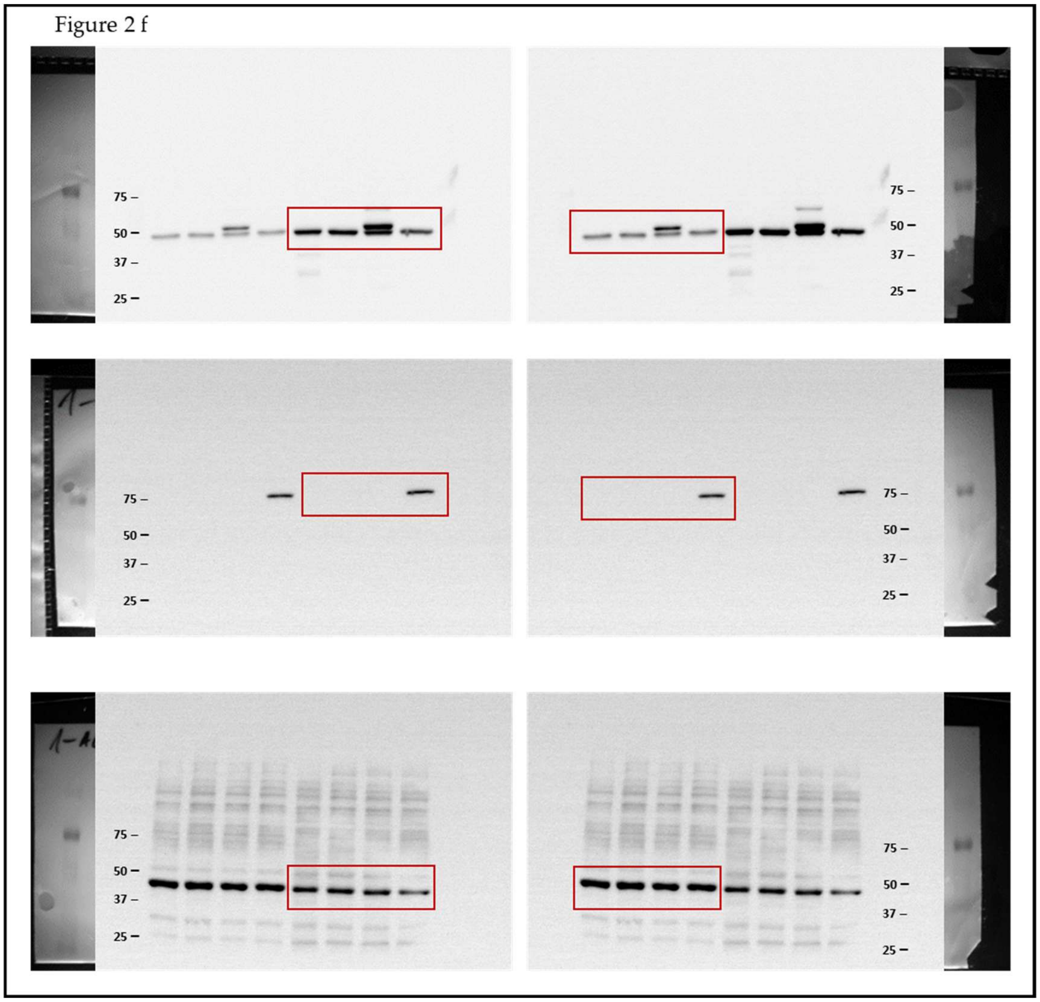

(f)

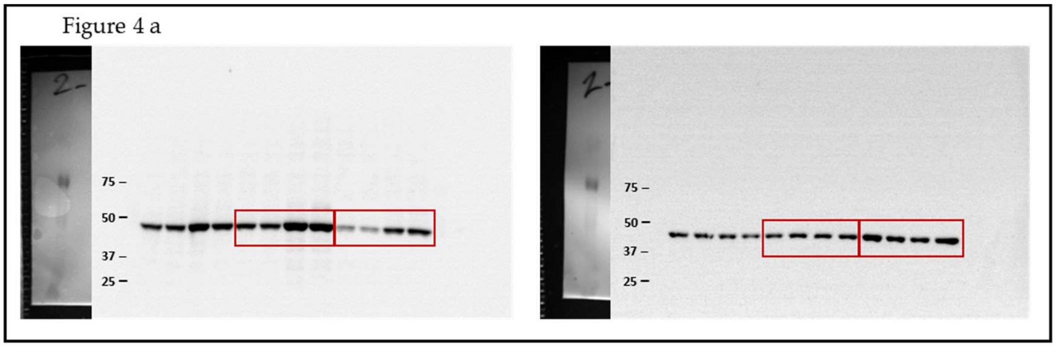

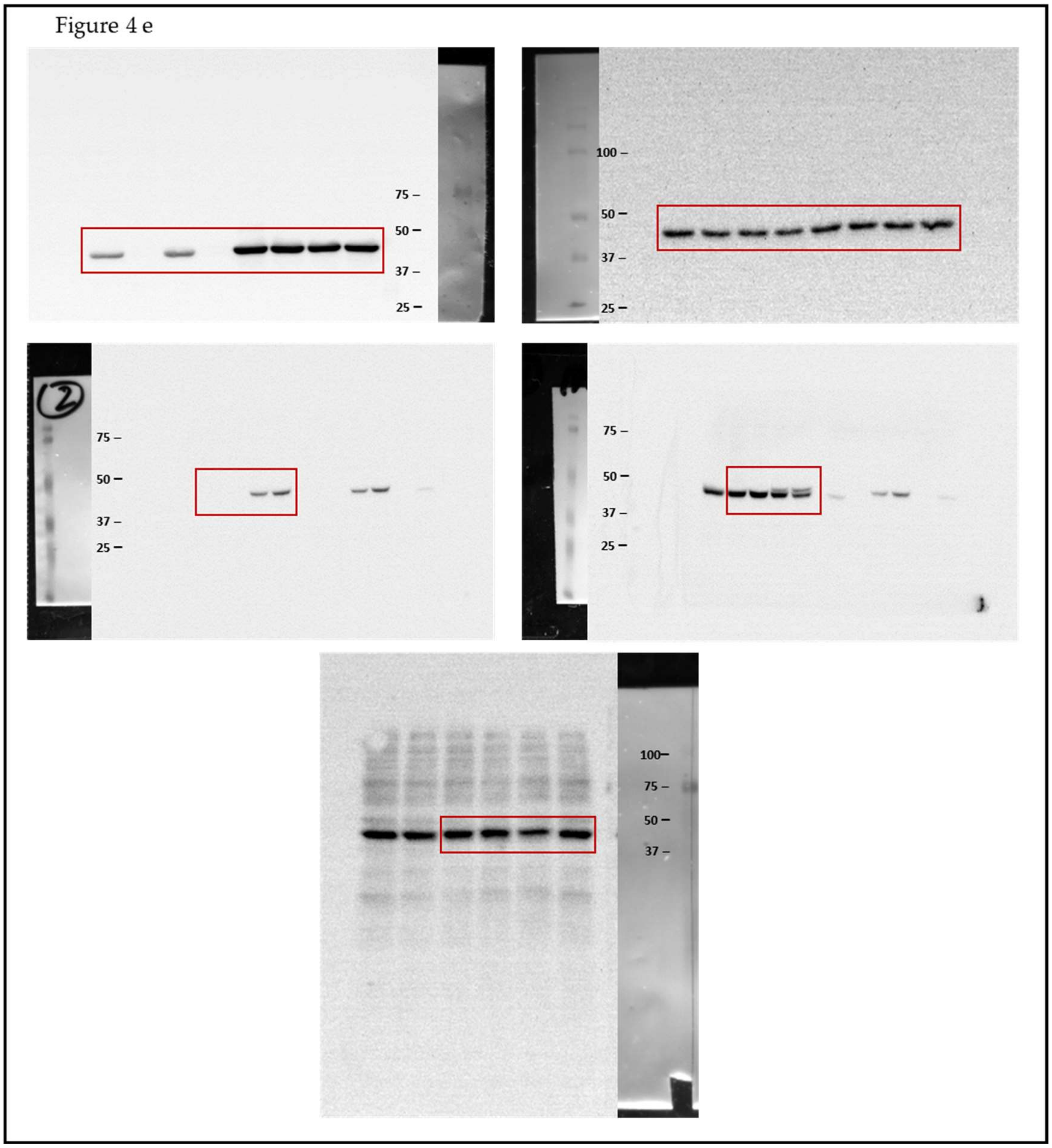

cancers MDPI

(g)

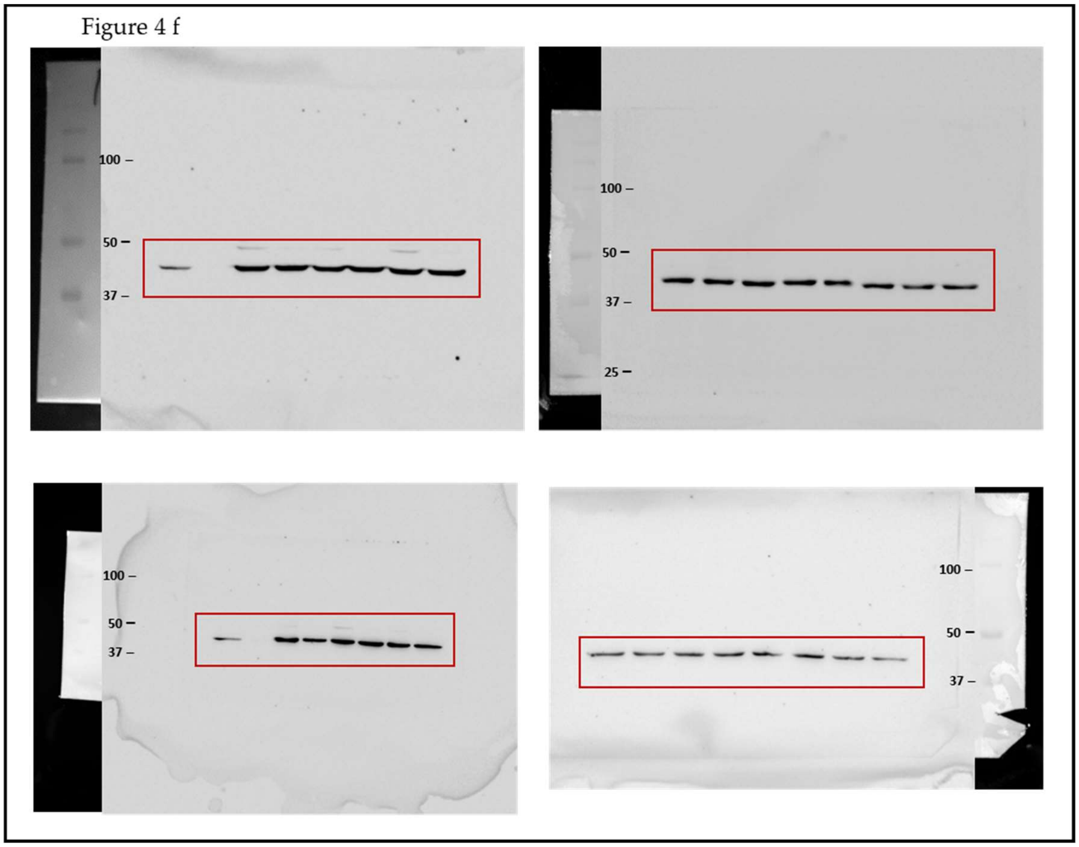

Figure S7. Full-length western blots presented in the manuscript. **(a) (b) (c) (d) (e) (f) (g)** Western blots presented in the indicated figures.
